# 24‐hour activity cycle movement behaviours and brain volume in people with mild cognitive impairment: A compositional and isotemporal substitution analysis

**DOI:** 10.1002/alz.091856

**Published:** 2025-01-09

**Authors:** Guilherme Moraes Balbim, Nárlon Cássio Boa Sorte Silva, Ryan S Falck, Teresa Liu‐Ambrose

**Affiliations:** ^1^ Vancouver Coastal Health Research Institute, Vancouver, BC Canada; ^2^ University of British Columbia, Vancouver, BC Canada; ^3^ Centre for Aging SMART, Vancouver, BC Canada; ^4^ Djavad Mowafaghian Centre for Brain Health, Vancouver, BC Canada; ^5^ Western University, London, ON Canada; ^6^ Centre for Aging SMART, Vancouver Coastal Health Research Institute, Vancouver, BC Canada; ^7^ Lawson Health Research Institute, London, ON Canada

## Abstract

**Background:**

People with mild cognitive impairment are at greater risk of Alzheimer’s disease. Physical activity (PA), sedentary behaviour, and sleep are movement behaviours constituting the 24‐hour activity cycle (24‐HAC) and are interactively associated with cognitive and brain health. However, the relationship between 24‐HAC compositions (i.e., time in one behaviour relative to remaining) and brain volume in this population remains underexplored. We aimed to investigate the associations between 24‐HAC compositions and time reallocation of 24‐HAC movement behaviours with brain volume.

**Method:**

A cross‐sectional study in 110 community‐dwelling adults (55+ years) with mild cognitive impairment (Montreal Cognitive Assessment <26/30). MotionWatch8© assessed 24‐HAC movement behaviours (5‐7 days). Compositional data analysis determined 24‐HAC compositions (four isometric log‐ratio pivot coordinates). FreeSurfer quantified gray matter volume using T1‐weighted magnetic resonance imaging. Linear regressions adjusted for age, sex, intracranial volume, and education determined 24‐HAC compositions and brain volume associations. Compositional isotemporal substitution analysis estimated the difference in brain volume associated with reallocating time between pairs of 24‐HAC movement behaviours. The Benjamini‐Hochberg false‐discovery rate (FDR) adjusted *p*‐values for multiple comparisons.

**Result:**

Higher moderate‐to‐vigorous PA composition was associated with greater cortical volume in the inferior temporal gyrus TE2a region (ß = 0.30, 95% CI = 0.15; 0.44, FDR‐adjusted‐*p* = 0.030). Higher light PA composition was associated with lower cortical volume in the TE2a region (ß = ‐0.45, 95% CI = ‐0.65; ‐0.24, FDR‐adjusted‐*p* = 0.015). Sedentary behaviour and sleep were not associated with brain volume (FDR‐adjusted‐*p*>0.05). Reallocating 30 minutes from sedentary behaviour to moderate‐to‐vigorous PA was associated with 2.1% greater volume in the TE2a region (ß = 0.06, 95% CI = 0.02; 0.10, *p*<0.001). Reallocating 30 minutes from moderate‐to‐vigorous PA to sedentary behaviour was associated with 2.8% lower volume in the TE2a region (ß = ‐0.08, 95% CI = ‐0.14; ‐0.02, *p*<0.001).

**Conclusion:**

Greater light PA composition may happen at the expense of lower moderate‐to‐vigorous PA composition, which could explain its associations with lower cortical volume. Greater moderate‐to‐vigorous PA composition and reallocating time from sedentary behaviour to moderate‐to‐vigorous PA may counteract cortical atrophy in an Alzheimer’s disease signature region in people with mild cognitive impairment.

